# The D ~ Sense ex-vivo viability assay application in a patient with stage IV lung adenocarcinoma: a case report

**DOI:** 10.1186/s13256-023-04277-2

**Published:** 2023-12-24

**Authors:** Yu Zhang, Xiaoyuan Wu, Ping He, Jieyu Wu, Xia Gu, Matyas Bendek, Rita Ötvös, Laszlo Szekely

**Affiliations:** 1 Nanjing Chest Hospital, Nanjing, 210029 China; 2 Department of Pathology, The First Affiliated Hospital of Guangzhou Medical University, Guangzhou, 510230 China; 3 Department of Microbiology, Tumor and Cell Biology, Karolinska Institutet, 17165 Stockholm, Sweden; 4 Department of Pathology/Cytology, Karolinska University Laboratory, Karolinska University Hospital Huddinge, Huddinge, 14186 Stockholm, Sweden

**Keywords:** Non-small lung cancer, Drug resistance, Ex-vivo viability assay, Combination therapy, Case report

## Abstract

**Background:**

The treatment resistance is a problem for lung cancer. In this study, we used a vitro tissue culturing system to select a new therapy strategy for a patient with tyrosine kinase inhibitors (TKIs) resistance.

**Case presentation:**

A 42-year-old male Asian patient was diagnosed with advanced lung adenocarcinoma harboring an exon 19 deletion in the epidermal growth factor receptor (EGFR) gene. The patient was treated with Gefitinib, resulting in an almost complete remission for over a year. The patient relapsed after 13 months treatment, and received four cycles of chemotherapy. At 20 months, the patient had developed multiple lung metastases and a solitary cerebellar metastasis. An EGFR T790M mutation was identified in the peripheral blood sample. Subsequent treatment with Osimertinib resulted in a complete response of the intracranial metastasis. By 33 months, the patient had developed a mediastinal tumor mass that responded well to local radiotherapy. By 39 months, an EGFR C797S cis-mutation had been identified and the patient was treated with Brigatinib and Cetuximab. By 44 months, the tumor cells from the pleural effusion had been tested for sensitivity against 30 targeted and cytostatic drugs using the D ~ Sense ex-vivo viability assay. The assay identified 8 drugs with moderate to high sensitivity. Combination therapy of Gemcitabin and Lobaplatin had resulted in disease stabilization.

**Conclusions:**

The case showed that individualized treatment aided by D ~ Sense ex-vivo viability assay can be a viable option for patients with advanced lung adenocarcinoma with pleural effusions.

**Supplementary Information:**

The online version contains supplementary material available at 10.1186/s13256-023-04277-2.

## Background

The introduction of molecular targeted therapy of oncogenic tyrosine kinases has fundamentally changed the diagnosis and therapy of lung adenocarcinomas [1]. Identification of the relevant mutations and the use of matching targeted small molecular inhibitors has created a new paradigm in the clinical workflow [2]. Unfortunately, the rapid emergence of tumor cells with escape mutations during therapy sets the limits for the usefulness of the technology [3]. Patients with no targetable mutations or with confirmed resistant mutations are treated with chemotherapy [4].

The treatment options for drug-resistant tumors are limited [5]. Spreading of the tumor into the pleural space confers worsens the disease prognosis [6]. The emergence of tumor cells in the effusion fluid provides a new opportunity for diagnostic characterization of the drug sensitivity of the most malignant variants of the tumor cells [7, 8]. High-throughput automated ex-vivo testing viable tumors from pleura or other effusion against various drug arrays has recently become an option to provide clinically relevant information about the actual drug sensitivity of the tumor cells against a large panel of drugs [9].

Here, we demonstrate that selecting a combination therapy based on the measurement of ex-vivo killing effect of a 30-drug panel in a 6–9 day-long survival assay resulted in a clinical benefit for a stage IV lung adenocarcinoma patient with multiple EGFR escape mutations and a refractory phenotype for conventional chemotherapy and radiotherapy.

## Case presentation

**Fig. 1:**
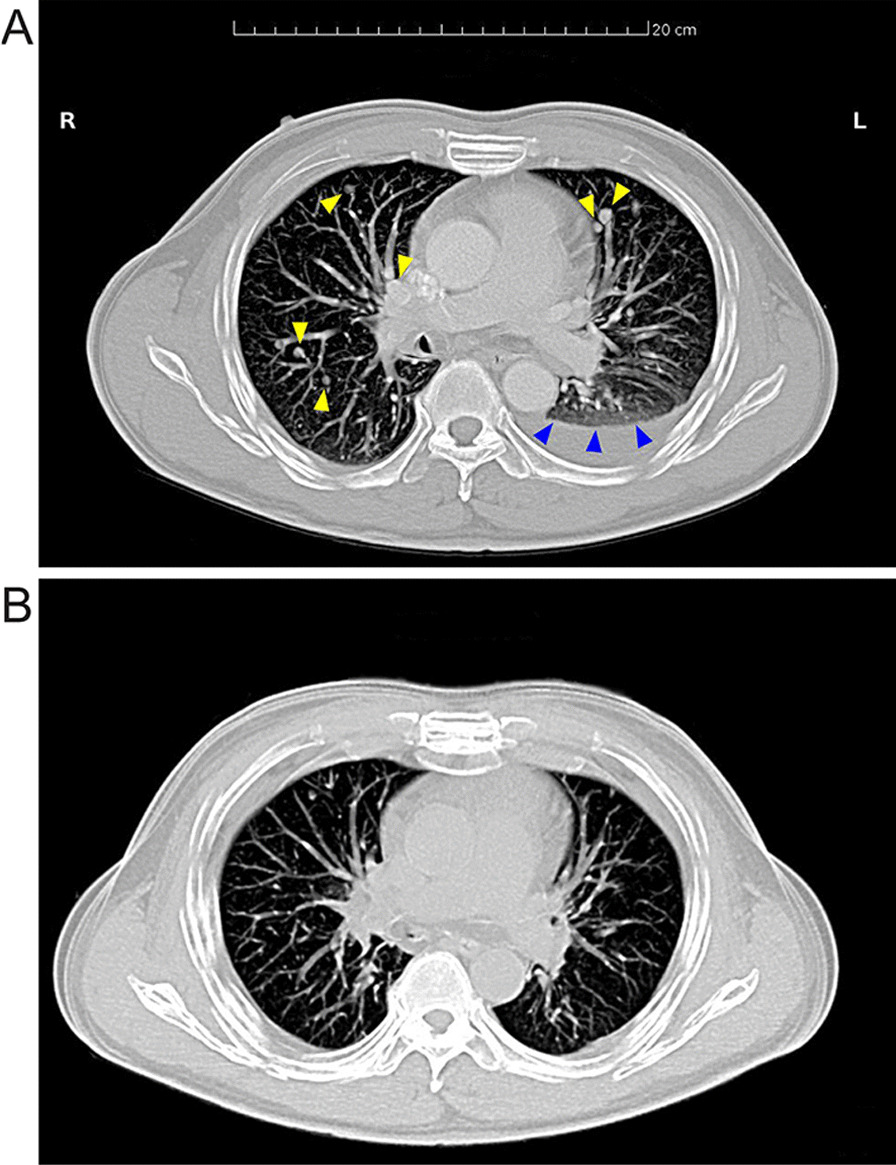
The chest CT images of the patient before and after Gefitinib treatment. **A** The patient’s chest CT image at the initial diagnosis showed multiple parenchymal tumor nodules (yellow arrows). Lymph nodes were affected as well as the accumulation of effusion fluid in the left pleural cavity (blue arrows). The patient was categorized as a stage c-T2N2M1. **B** The chest CT image of the patient after receiving Gefitinib treatment for 13 months

**Fig. 2:**
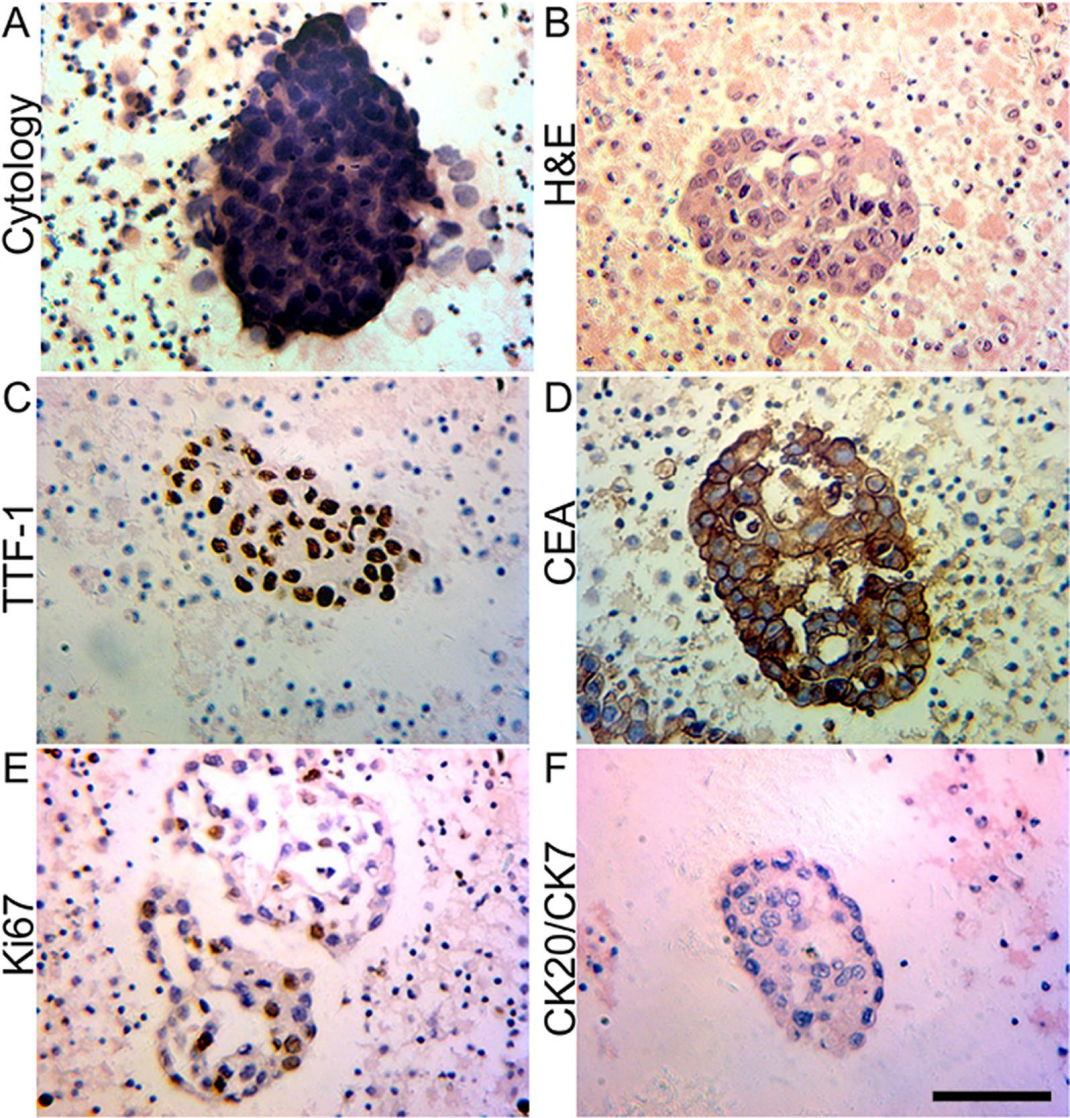
H&E and immunohistochemistry staining of the tumor cell clusters in the pleural effusion. **A** Cytology of the smear of the effusion (MGG). **B** H&E staining of the cell block of the effusion. **C** and **D** TTF-1 and CEA positive staining of the tumor cells. **E** Ki67 staining to show the proliferated tumor cells. **F** CK20 and CK7 double-staining were negative in the tumor cells. Bar = 50 μm

A 42-year-old male, non-smoking Asian patient sought medical help with chest pain and perceived tightness in the left chest. He had no family history of cancer. Upon physical examination, percussion dullness and decreased respiratory sound were noted in the left lower lung. Laboratory examination showed no sign of hematuria or blood in the feces. The C-reactive protein (CRP), blood coagulation, electrolytes, liver and kidney functions and fasting blood glucose were all within the reference range. The serum tumor markers, including cytokeratin 19 fragment (CYFRA21-1), neuron-specific enolase (NSE) and squamous cell carcinoma antigen (SCC) were all within reference range, while carcinoembryonic antigen (CEA) had a slight elevation. Chest CT revealed that diffuse infiltration in the left lower lobe, as well as effusion in the left side pleural and multiple nodes, were found in both lungs (Fig. 1A). Non-small tumor cells were found by histological analysis of the drained thoracic effusion (Fig. 2A). Immunohistochemical staining showed that the tumor cells were positive for thyroid transcription factor 1 (TTF-1) and CEA, but negative for prostate-specific antigen (PSA), Wilms tumor 1 (WT-1), p40, cytokeratin 5/6 (CK5/6), CK7, CK20 and anaplastic lymphoma kinase (ALK). Ki67 staining revealed a proliferation rate of approximately 38%. The H&E and immunohistochemistry results confirmed the tumor cells originated in the lung and were consistent with a poorly differentiated adenocarcinoma (Fig. 2B–F). Further molecular analysis of tumor tissue by the amplification refractory mutation system (ARMS)—PCR demonstrated an exon 19 deletion of the epidermal growth factor receptor (EGFR) gene (Table 1). The final diagnosis of this patient was adenocarcinoma of the lower left lung, T2N2M1, Stage IV, PS score (see vol. 1 in [11]).

**Table 1:** The initial detection of EGFR and p53 mutations by next generation sequencing in plasma and pleural effusion

Gene name	Mutation	Mutation type	Plasma abundance	Pleural effusion abundance
EGFR	p.E746_A750del No.19 Exon non-frameshift deletion mutation	c.2235_2249delGGAATTAAGAGAAGC(p.E746_A750del)	1.7%	22.2%

For treatment, the Gefitinib (250 mg, QD) was initiated, resulting in an almost complete remission within six weeks (Fig. 1B). The patient remained symptom free for over a year. By thirteen months, however, multiple solid metastatic lesions appeared in both lungs on chest CT. A peripheral-blood-based PCR was performed but did not identify other mutations. Subsequently, the patient received four cycles of Pemetrexed (800 mg, Day 1) and Cisplatin (40 mg, Day 1 to 3), resulting in disease stability for an additional three months.

**Fig. 3:**
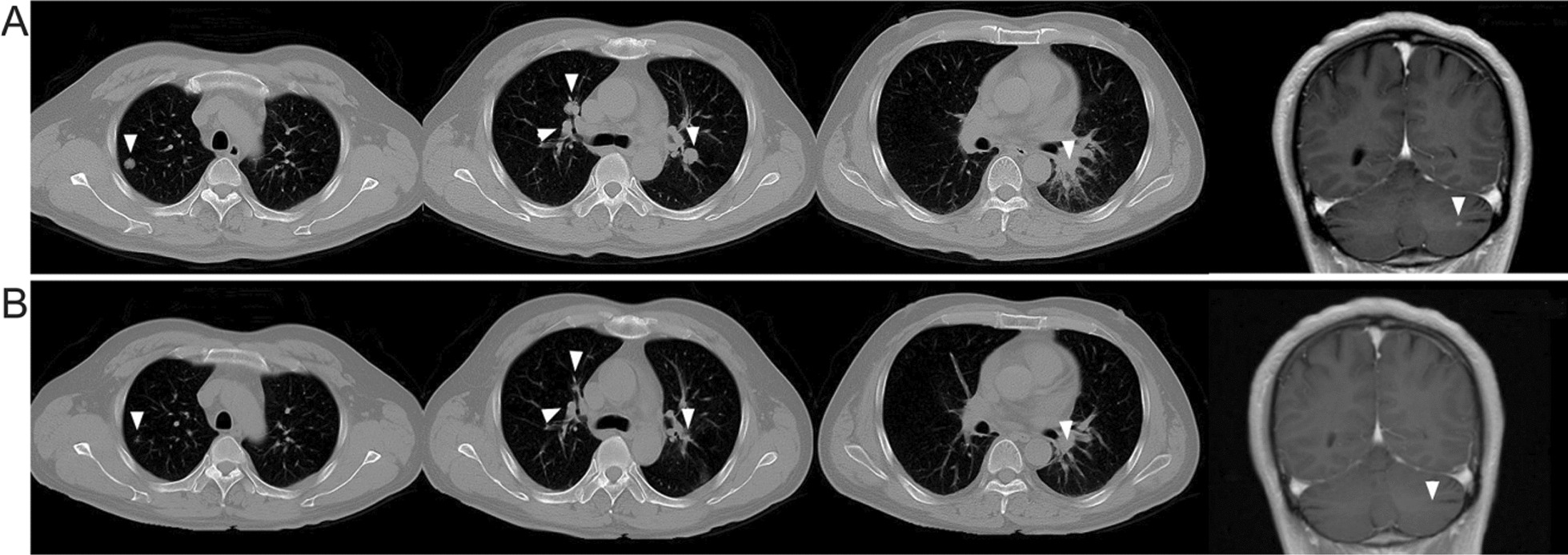
The chest and brain CT scan of before and after Osimertinib treatment. **A** Multiple pulmonary nodules and a cerebellar lesion had appeared in the CT scan, which indicated disease relapsed at 20 months after the initial diagnosis (white arrows). By this time the patient also received additional chemotherapy of Pemetrexed and cisplatin. EGFR-T790M is detectable in the peripheral blood. **B** Osimertinib treatment resulted in a partial response of the pulmonary lesions and complete response of the cerebellar lesion.

At 20 months, a CT scan of the patient revealed multiple metastases in both lungs and a metastatic lesion in the left cerebellar hemisphere (Fig. 3A). Molecular analysis of the peripheral blood identified an EGFR T790M mutation, for which the patient was given Osimertinib treatment (80 mg, QD). This resulted in complete response of the intracranial metastasis and partial response of the lung lesions (Fig. 3B).

**Fig. 4:**
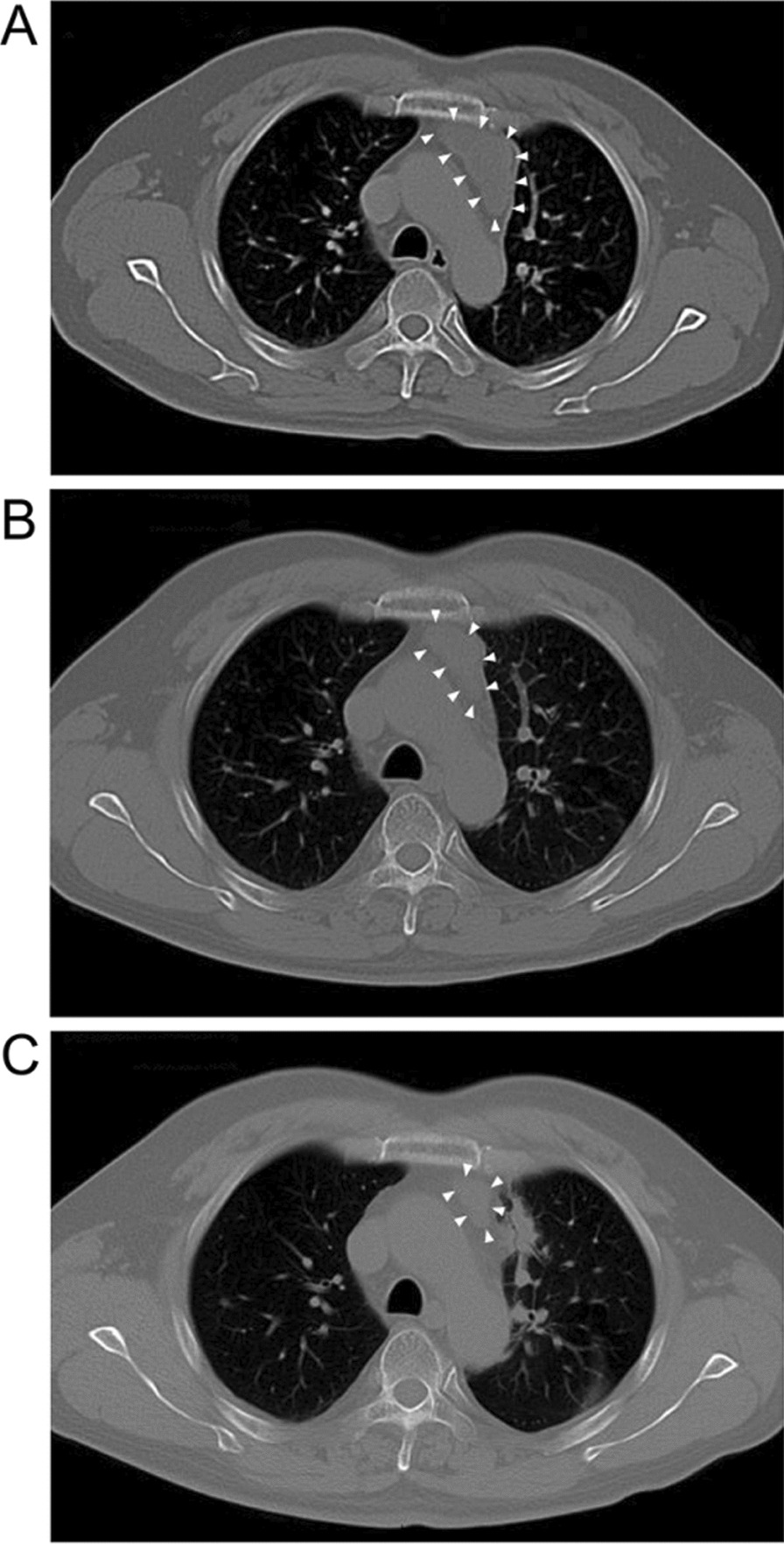
Emergence of mediastinal mass was partial response to local radiotherapy. **A** A mass was noticed in mediastina (white arrows). **B** and **C** It showed partial response to local radiotherapy (white arrows)

At 33 months, a CT scan revealed a mediastinal mass, which was biopsied and found to be adenocarcinoma. The patient then received local radiotherapy (DT: 60 Gy, in 20 fractions), resulting in a tumor volume decreased by over 80% (Fig. 4).

**Fig. 5:**
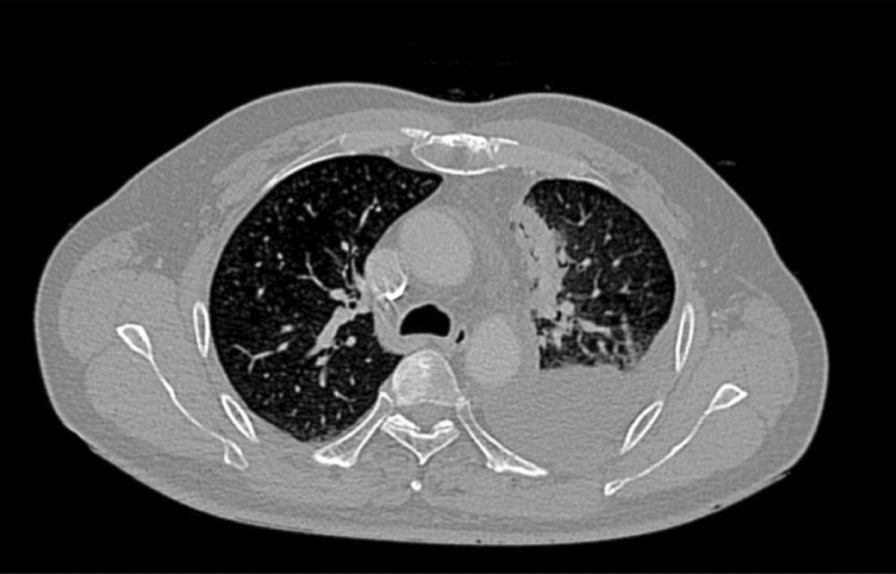
The chest CT scan showed pleural effusion and mass expansion. Accumulation of pleural effusion and expansion of solid mass in the left thorax by 39 months

**Fig. 6:**
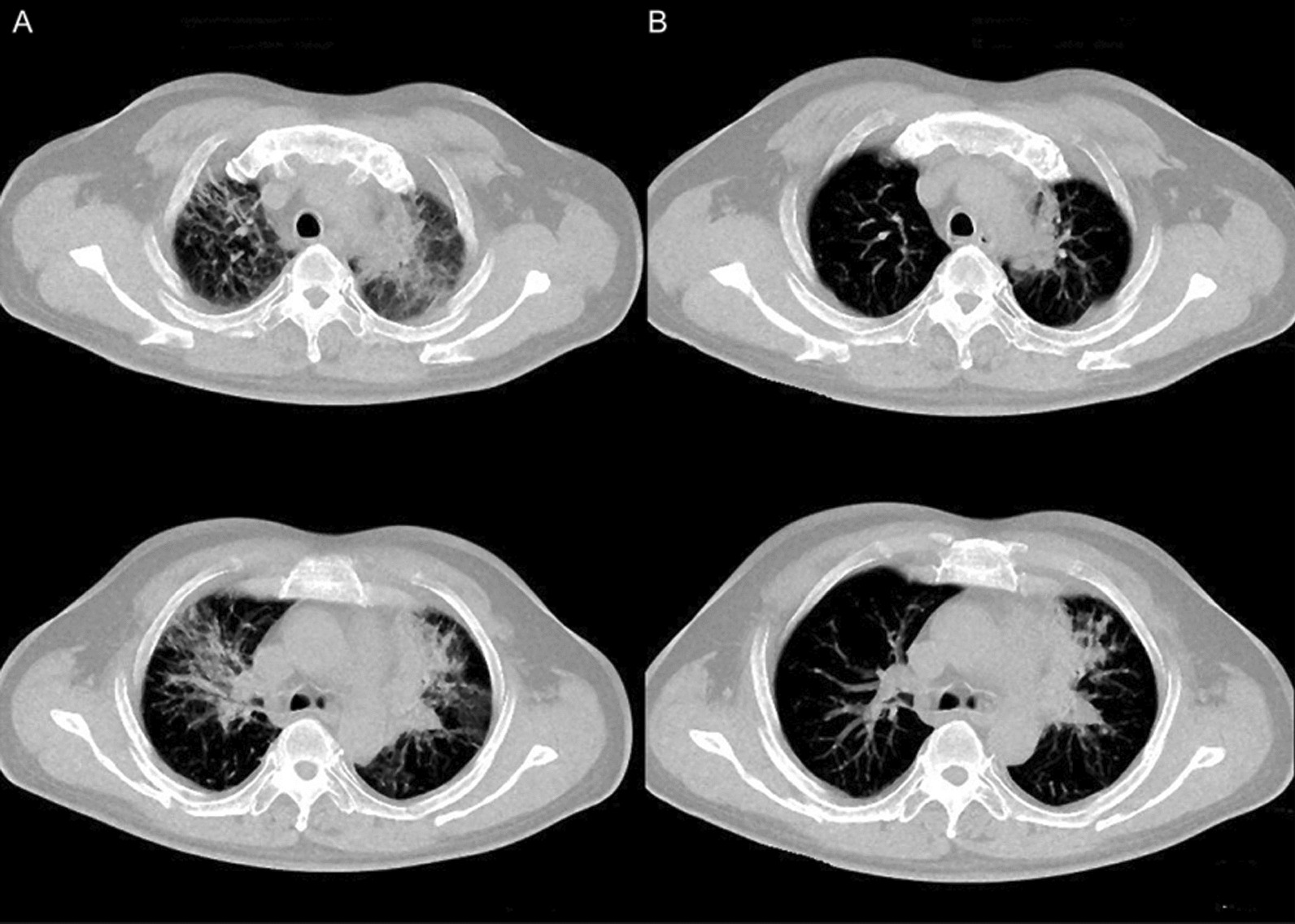
The chest CT scan demonstrated pneumonia of Brigatinib. **A** The Brigatinib treatment induced interstitial pneumonia. **B** The treatment with methylprednisolone was performed and pneumonia was resolved

By 39 months, a solid mass and pleural effusion had developed in the left thorax (Fig. 5). A next generation sequencing (NGS) analysis of the cells from the pleural effusion revealed multiple additional EGFR mutations, including 2389 T > A and 2390 G > C, both causing C797S amino acid substitution, and 2386 G > A causing C796S substitution (Table 2). To counter the effect of these mutations, the patient received Brigatinib and Cetuximab treatment. However, the patient developed interstitial pneumonia (Fig. 6A) after treatment. Steroid treatment was used and successfully resolved interstitial pneumonia (Fig. 6B).

**Table 2:** The secondary detection of EGFR and p53 mutations by next generation sequencing in plasma and pleural effusion

Gene name	Mutation	Mutation type	Plasma abundance	Pleural effusion abundance
EGFR	p.T490M No.20 exon Missense mutation	c.2369C > T (p.T790M)	1.4%	8.5%
EGFR	p.C797S No.20 Cis-missense mutation	c.2389T > A (P.T797S)	0.8%	–
EGFR	p.C797S No.20 Cis-missense mutation	c.2390G > C (p.C797S)	0.5%	8.6%
EGFR	p.C797S No.20 Cis-missense mutation	c.2386G > A (p.C796S)	–	0.4%
EGFR	p.C797S No.20 Cis-missense mutation	c.2360_2361delAginsGA (p.Q787R)	–	8.1%
EGFR	p.Q192*No.6 exon truncating mutation	c.574C > T (p.Q192*)	–	6.3%

*EGFR* Epithelial growth factor receptor, *A* adenine, *G* guanine, *T* thymine amino acid code for mutation *M* methionine, *S* serine, *T* theronine, *Q* glutamine, *C* cysteine, *R* arginine *STOP codon

**Fig. 7:**
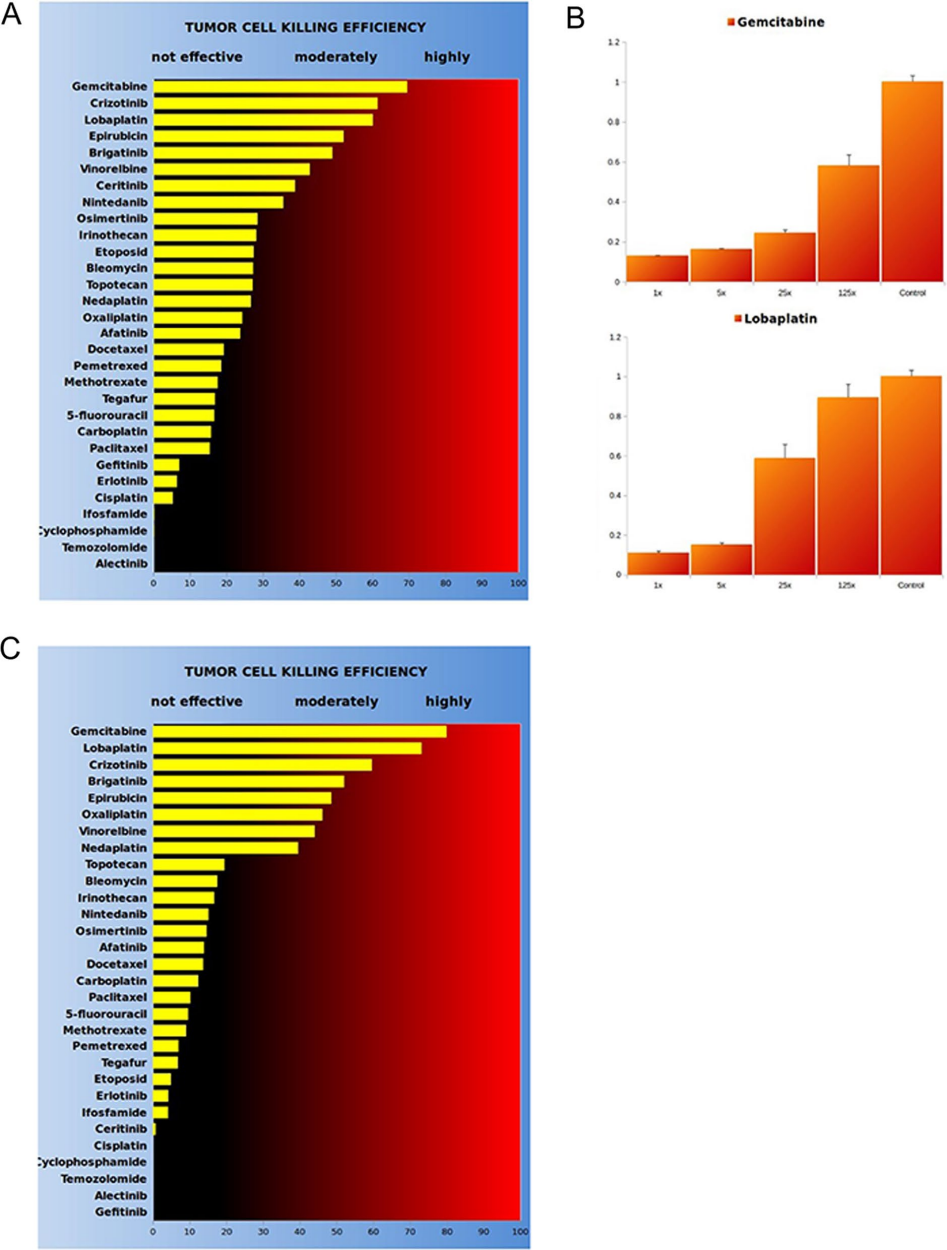
D~Sense ex-vivo viability assay was performed and identified new strategy of combination therapy. **A** and **C** The isolated tumor cells from the effusion were conducted ex-vivo survival test under different concentration of multiple drugs. The ranking shows the killing efficiency (KE%). **B** The ratio of viable cells in different drug concentration were detected

**Fig. 8:**
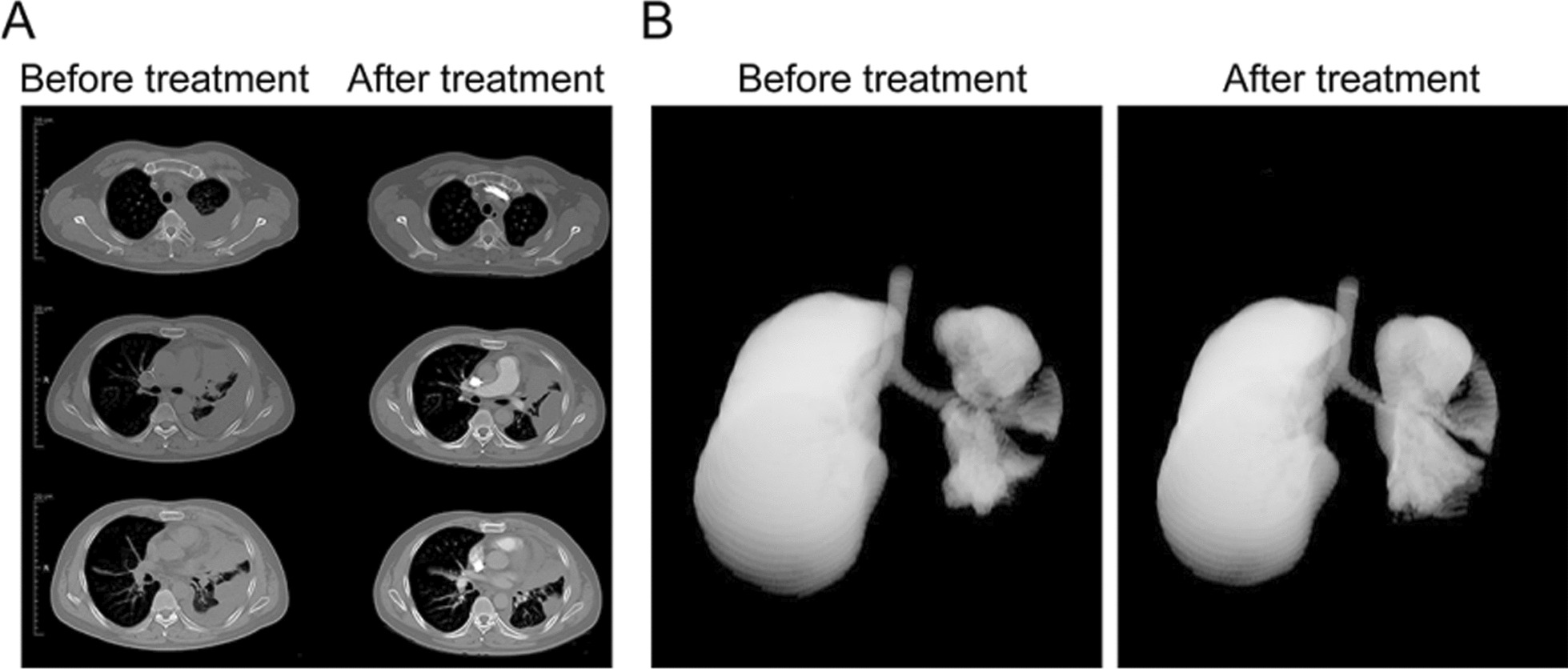
Therapeutic effects of the drugs selected by D~Sense assay in patient. **A** CT scan images before and after the combination therapy of Gemcitabine and Lobaplatin. **B** The 3D reconstituted lung airspace images before and after the combination therapy of Gemcitabine and Lobaplatin. showing a 16% increase of volume after therapy

By 42 months, the tumor cells from the pleural effusion were tested for sensitivity against 30 targeted and cytostatic drugs using the D ~ Sense ex-vivo viability assay (Fig. 7 and Additional file 1: Table S1). The assay identified eight drugs with moderate to high sensitivity. The Lobaplatin and Vinorelbin were chosen for combination therapy. However, this set of drugs was unable to control the pleural effusion. A repeated assay by months 44 identified the similar set of most effective drugs, whereupon selection of the two most effective drugs, Gemcitabin and Lobaplatin (Fig. 7B, C and Additional file 1: Figs. S1 and S2), resulted in full symptom control, reduction of pleural effusion, and stable disease for the solid lesions (Fig. 8A) as well as a 16% increase of the air capacity of the left lung (Fig. 8B).

## Discussion and conclusions

Identification and targeting cancer driver mutations has become the cornerstone of modern therapy for lung adenocarcinomas [12]. Despite the rapid progress in molecular diagnostic procedures and the emergence of new mutation-specific targeted drugs, relapse after successful temporary disease control is more the rule than an exception. Treating a genetically diverse tumor with single pharmacological agents has a very high likelihood of selecting out resistant variants, thus rendering even the most sophisticated targeted therapy ineffective in the long run.

An additional option to find potentially effective drugs is to directly expose isolated tumor cells in ex-vivo assays [13]. D ~ Sense assay is an improved, fully automated version of a clinical diagnostic assay that we have developed during the past decade [14–17]. The assay provides highly reproducible, robust measurement of drug-induced cytotoxicity on a single cell level using triplicates of the four-step drug dilution series. The drugs are tested in a concentration range that is proportional to the clinical dose and achievable in the in vivo tumor tissue. The 384 well pre-printed drug plates allow for simultaneous testing and ranking of 30 anti-cancer agents. A unique feature of the assay is that it is performed in a hermetically closed, protein-rich, reducing environment that closely mimics the hypoxic interstitial fluid of the tumor tissue. The viability detection is based on the fluorescence detection of intact chromatin. The fixation, staining and quenching for unbound fluorescence is performed in a single step without the need of washing or fluid exchange. D ~ Sense assay applies for samples where the tumor cells are growing in suspension form, such as leukemias, lymphomas, and tumor cells in serous effusions. The assay is intended for patients who have exhausted conventional and targeted protocols. Tumors undergo intricate genetic and epigenetic evolution, which consistently leads to variant drug resistance. These variances can differ in genetic composition at different sites. Considering the adaptive tumor cells have the ability to freely invade in the body cavity effusions. The fast proliferating cancer cells in these fluids can be considered the pinnacle of cancer progression, which with a representation of more and more aggressive phenotype. With the primary site tissue histology diagnosis, we used effusion cytology to testify the tumor cells with aggressive behaviour than their counterparts still growing in solid forms.

**Fig. 9:**
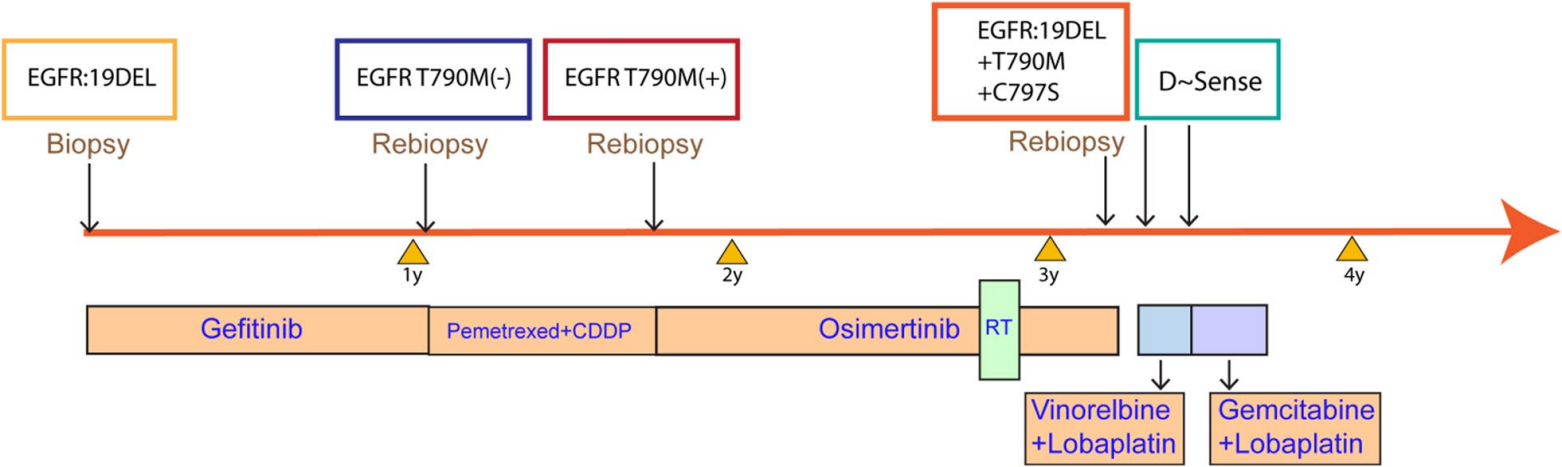
The clinical timeline of the case

Here, we demonstrate successful disease control for a period of almost four years in a patient with stage IV lung adenocarcinoma (Fig. 9). Initially, therapy was guided by targetable mutations of EGFR, and a successive series of three TK inhibitors (gefitinib, osimertinib, brigatinib) were employed in adaptation to the emergence of escape mutations. In the absence of molecular guidance, conventional chemotherapy (pemetrexed and cisplatin), anti-angiogenesis therapy (cetuximab) as well as focal irradiation were used.

When all options of mutation based targeted therapy were exhausted, the ex-vivo drug sensitivity testing provided additional treatment possibilities. The combination of the two most effective drugs resulted in a reduction in the tumor mass and an increase in the lung aeration. In addition, full symptom control was achieved. However, we did not further follow up the patient after his symptom was fully controlled in this study. We have an ongoing case series study to continue to use this assay in patients with different tumor in advanced stages. The follow-up study is conducting in this series of patients. Also, in this one case report, we could not quantify the correlation between the drug sensitivity after D ~ Sense scanning and the treatment effect. We need to include larger patient population to confirm that the drugs selected by D ~ Sense assay is effective in most patients. Further follow-up studies, including progression free survival and overall survival will be conducted to resolve the limitations in this study.

The D ~ Sense is a highly throughout and replicable drug sensitivity assay for tumor patients. With this customized treatment selection system, patients who have exhausted conventional and targeted protocols could receive practicable treatment.

### Supplementary Information

**Additional file 1: Fig. S1.** The digitally processed images of Lobaplatin on the D~Sense drug system. The composited images showed the effect of Lobaplatin on the series of digitally processed images (top) and the raw images from selected wells treated with the highest (left) and lowest (right) drug concentrations. **Fig. S2**. The assay reliability report. The assay reliability scores illustrating the robustness of the measurements and calculations. **Fig S3.** The digitally processed images of the 384 wells of the D~Sense drug plate. **A** Zoomable mosaic of the digitally processed images of the 384 wells of the D~Sense drug plate showing the fluorescence signal of the surviving cells on triplicate lines of 4 steps drug dilutions. **B** Thirty tested drugs and concentration (μM). **Table S1.** The drug concentrations (μM) of 30 candidates were used in the assay.

## Data Availability

All the drug sensitivity measurement data including the raw and processed images, the two levels zoom mosaics, the drug titration charts and killing efficiency rankings are available through contacting the corresponding author.

## Abbreviations

ARMS-PCR: Amplification refractory mutation system-polymerase chain reaction
ALK: Anaplastic lymphoma kinase
CEA: Carcinoembryonic antigen
CRP: C-reactive protein
CK: Cytokeratin
CT: Computer tomography
CYFRA21-1: Cytokeratin 19 fragment
EGFR: Epithelial growth factor receptor
H&E: Hematoxylin–eosin staining
MGG: May-Grunwald Giemsa staining
NSE: Neuron specific enolase
PSA: Prostate-specific antigen
PS score: Scale of performance status
QD: Once a day
SCC: Squamous cell carcinoma antigen
TK: Tyrosine kinase
TTF-1: Thyroid transcription factor 1
WT-1: Wilms tumor 1
