# Supplementary material for: The D ~ Sense ex-vivo viability assay application in a patient with stage IV lung adenocarcinoma: a case report
Source: J Med Case Rep. 2023 Dec 24;17:529. doi: 10.1186/s13256-023-04277-2 (PMC10748857; doi:10.1186/s13256-023-04277-2)
Supplement: Supplementary file 1 — Additional file 1: Fig. S1. The digitally processed images of Lobaplatin on the D~Sense drug system. The composited images showed the effect of Lobaplatin on the series of digitally processed images (top) and the raw images from selected wells treated with the highest (left) and lowest (right) drug concentrations. Fig. S2. The assay reliability report. The assay reliability scores illustrating the robustness of the measurements and calculations. Fig S3. The digitally processed images of the 384 wells of the D~Sense drug plate. A Zoomable mosaic of the digitally processed images of the 384 wells of the D~Sense drug plate showing the fluorescence signal of the surviving cells on triplicate lines of 4 steps drug dilutions. B Thirty tested drugs and concentration (μM). Table S1. The drug concentrations (μM) of 30 candidates were used in the assay. [file 13256_2023_4277_MOESM1_ESM.docx]

**Additional file**

**The D~Sense ex-vivo viability assay application in a patient with stage IV lung adenocarcinoma**

Yu Zhang^1^, Xiaoyuan Wu^1^, Ping He^2^, Jieyu Wu^2,3^, Xia Gu^2^, Matyas Bendek^4^, Rita Ötvös^4^, Laszlo Szekely^4*^

^1^ Nanjing Chest Hospital, Nanjing, 210029, China,

^2^Department of Pathology, The First Affiliated Hospital of Guangzhou Medical University, Guangzhou, 510230, China

^3^Department of Microbiology, Tumor and Cell Biology, Karolinska Institutet, Stockholm, 17165, Sweden

^4^Department of Pathology/Cytology, Karolinska University Laboratory, Stockholm, 14186, Sweden

**Correspondence:** *Correspondence, gallery proofs and reprint requests should be primarily addressed to Laszlo Szekely MD, PhD, Department of Pathology/Cytology, Karolinska University Hospital Huddinge, 141 86, Huddinge, Sweden. Email: laszlo.szekely@ki.se

**Materials and methods**

**Purification of the tumor cells**

Pleural effusion fluid was collected in sterile, heparinized plastic container. The cells of the effusion were enriched by centrifugation at 800g for 10 min and re-suspended in one-tenth of the original volume. Red blood cells and neutrophil granulocytes were removed by density gradient centrifugation on a lymphoprep cushion. Cells from the buffy coat layer were depleted for T lymphocytes using anti-CD3-coated magnetic beads. Contaminating macrophages and mesothelial cells were removed by selective adhesion to the bottom of collagen-coated tissue culture flask at 37°C for 30 min. The free-floating cells were collected, and their tumor origin was confirmed with cytology smears. The tumor cells were re-suspended in the proprietary D~Cult cell culture medium, which has similar reducing conditions and protein concentration as the effusion fluid.

**Measuring the ex-vivo cytotoxicity using the D~Sense Assay**

Approximate 3000 cells were plated in 30 μL volumes on a 384-well-plate containing 50 nL droplets of drugs in DMSO (for final drug concentrations, see Supplementary Fig. 3). All drugs were tested in four different concentrations in triplicates. The plates were sealed with airtight aluminum foil and incubated at 37°C for 6 and 9 days. Subsequently, the cells were fixed and stained with the proprietary D~Dye fluid that simultaneously fixes the cells, stains the intact chromatin of the viable cells, and quenches the unbound fluorescence. All fluid handling was performed using OT-2 laboratory robots running on custom-developed programs. The plates were analyzed using the D~Counter automated fluorescence imager that photographed the wells. A custom-developed image analysis program identified and counted the viable tumor cells using five independent imaging algorithms. The median value of the counting and the median value of the triplicates were used as a result of the assay for any concentration. The killing efficiency (KE%) was calculated as a weighted sum of all killings at the different concentrations, according to the formula we have previously published^10^.

The clinical reports, ranking of drug effects, concentration dependent survival charts and the fully zoomable mosaics of the raw and processed plate images were generated by custom-developed programs on the Linux computer.

**Additional file Figures and legends**


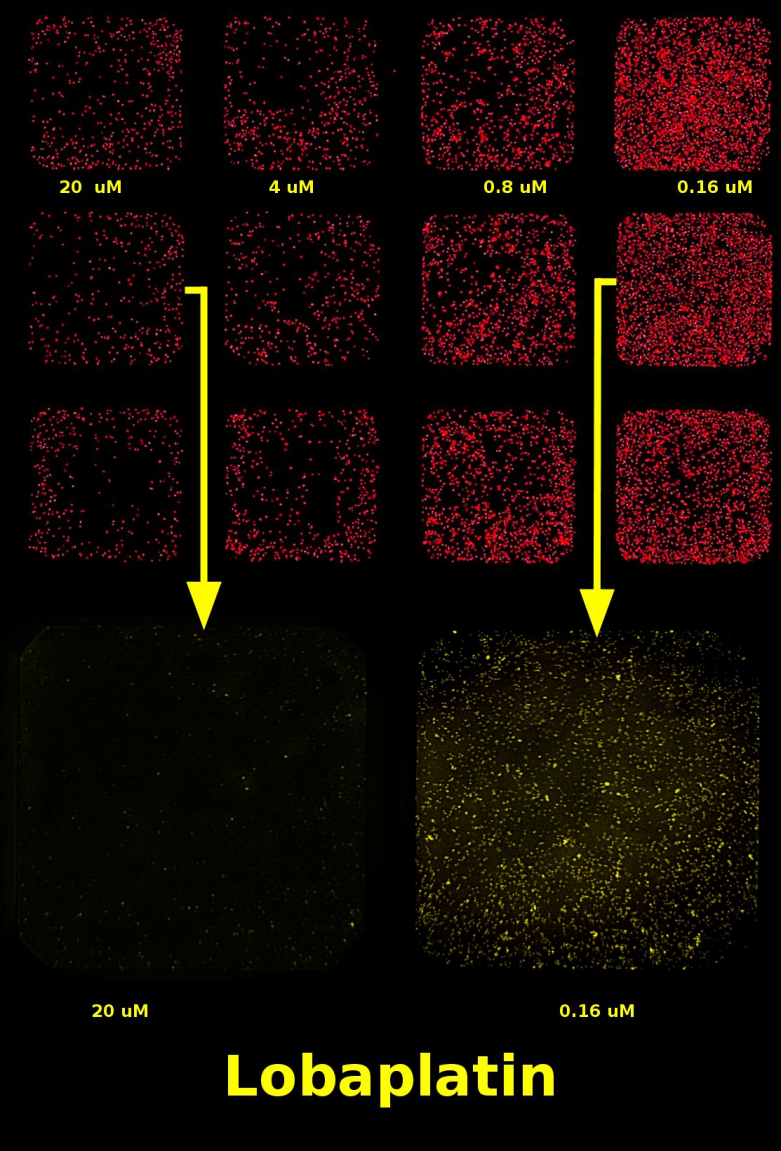


**Figure S1. The digitally processed images of Lobaplatin on the D~Sense drug system**

The composited images showed the effect of Lobaplatin on the series of digitally processed images (top) and the raw images from selected wells treated with the highest (left) and lowest (right) drug concentrations.


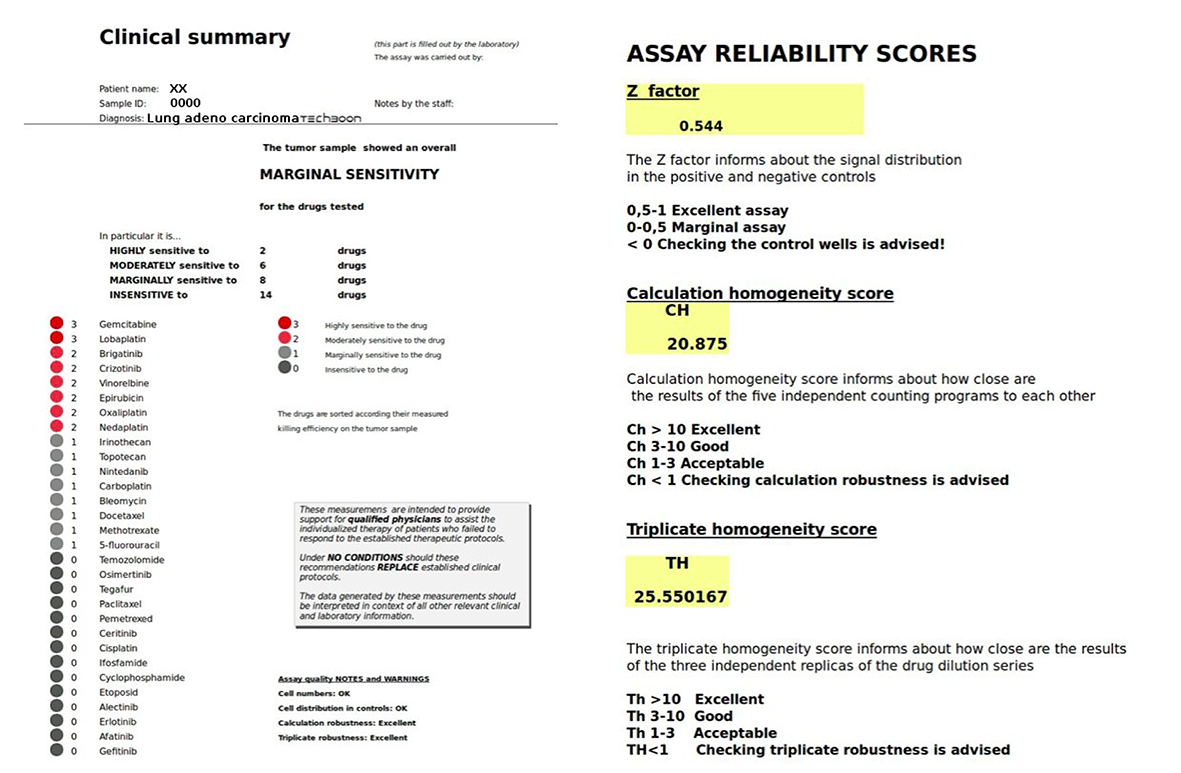


**Figure S2. The assay reliability report.**

The assay reliability scores illustrating the robustness of the measurements and calculations.

**
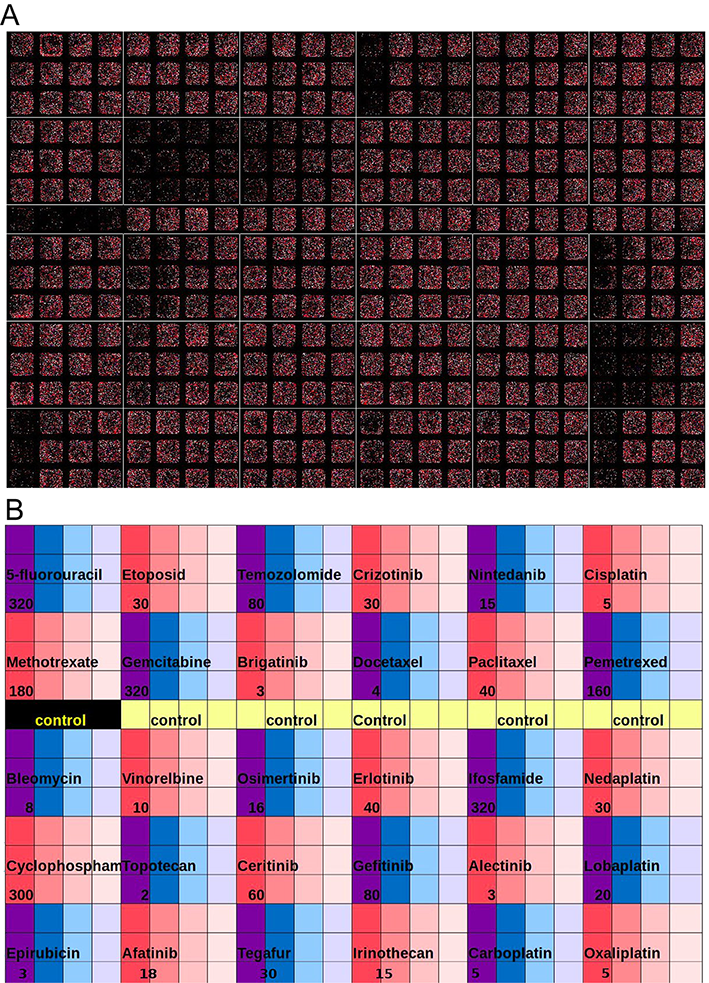
**

**Figure S3. The digitally processed images of the 384 wells of the D~Sense drug plate**

**A.** Zoomable mosaic of the digitally processed images of the 384 wells of the D~Sense drug plate showing the fluorescence signal of the surviving cells on triplicate lines of 4 steps drug dilutions.

**B.** Thirty tested drugs and concentration (μM).

**Table S1. The drug concentrations (μM) of 30 candidates were used in the assay**

| No. | Drug | Maximum concentration (μM) |
| --- | --- | --- |
| 1 | 5-Fluorouracil | 320 |
| 2 | Methotrexate | 180 |
| 3 | Bleomycin | 8 |
| 4 | Cyclophosphamide | 300 |
| 5 | Epirubicin | 3 |
| 6 | Etoposid | 30 |
| 7 | Gemcitabine | 320 |
| 8 | Vinorelbine | 10 |
| 9 | Topotecan | 2 |
| 10 | Afatinib | 18 |
| 11 | Temozolomide | 80 |
| 12 | Brigatinib | 3 |
| 13 | Osimertinib | 16 |
| 14 | Ceritinib | 60 |
| 15 | Tegafur | 160 |
| 16 | Crizotinib | 30 |
| 17 | Docetaxel | 4 |
| 18 | Erlotinib | 40 |
| 19 | Gefitinib | 80 |
| 20 | Irinothecan | 60 |
| 21 | Nintedanib | 15 |
| 22 | Paclitaxel | 40 |
| 23 | Ifosfamide | 320 |
| 24 | Alectinib | 3 |
| 25 | Carboplatin | 5 |
| 26 | Cisplatin | 5 |
| 27 | Pemetrexed | 160 |
| 28 | Nedaplatin | 30 |
| 29 | Lobaplatin | 20 |
| 30 | Oxaliplatin | 8 |
